# Sexual dimorphic synaptic dysfunction and cognition in a mouse model of Alzheimer’s disease

**DOI:** 10.1002/alz.090760

**Published:** 2025-01-03

**Authors:** Caleigh A. Findley, Samuel A McFadden, Tiarra Hill, Mackenzie R. Peck, Kathleen Quinn, Kevin N. Hascup, Erin R. Hascup

**Affiliations:** ^1^ Southern Illinois University School of Medicine, Springfield, IL USA

## Abstract

**Background:**

Glutamatergic neurotransmission plays an essential role in learning and memory. Previous studies support a dynamic shift in excitatory signaling with Alzheimer’s disease (AD) progression, contributing to negative cognitive outcomes. The majority of previous studies have relied heavily on male physiology when determining these alterations in AD mouse models. Here, we examine sex differences in neurotransmission and cognition using the APP^NL‐F^ mouse model of AD.

**Method:**

Male and female APP^NL‐F^ and control C57BL/6 mice underwent cognitive assessment using Morris water maze (MWM) and Novel Object Recognition (NOR) at 2‐4 and 18+ months of age. Afterwards, mice were anesthetized with isoflurane while determining basal, stimulus‐evoked, and clearance rates of extracellular glutamate measures in the dentate gyrus (DG), CA3, and CA1 hippocampal subregions.

**Result:**

Our data support impaired spatial cognition in aged male and female APP^NL‐F^ mice, but only aged females displayed recognition memory deficits compared to age‐matched control mice. Evoked glutamate release was elevated in the DG and CA3 of young APP^NL‐F^ male mice that declined with age compared to age‐matched control mice. Young female APP^NL‐F^ mice exhibited increased glutamate clearance in the CA1 that slowed with age compared to age‐matched control mice. Both sexes of APP^NL‐F^ mice exhibited decreased CA1 basal glutamate levels, but only males also showed CA3 reductions.

**Conclusion:**

Our findings confirm a sex‐dependent hyper‐to‐hypoactivation glutamatergic paradigm in APP^NL‐F^ mice. Additionally, we observed a sexually dimorphic biological aging process resulting in a more severe cognitive phenotype for female APP^NL‐F^ mice than males. This research mirrors that of human AD pathology and provides further evidence of divergent AD pathogenesis between sexes.

